# The effect of antimicrobial additives on the properties of dental glass-ionomer cements: a review

**DOI:** 10.1080/23337931.2018.1539623

**Published:** 2019-01-10

**Authors:** Tamer Tüzüner, Aleksandar Dimkov, John W. Nicholson

**Affiliations:** a Department of Paediatric Dentistry, Faculty of Dentistry, Karadeniz Technical University, Trabzon, Turkey;; b Department of Paediatric and Preventive Dentistry, Faculty of Dental Medicine, St Cyril and St Methodius University, Skopje, Macedonia;; c Bluefield Centre for Biomaterials, London, United Kingdom;; d Dental Physical Sciences, Institute of Dentistry, Barts & The London School of Medicine and Dentistry, Queen Mary University of London, London, United Kingdom

**Keywords:** Glass-ionomer, antimicrobial, mechanical properties

## Abstract

**Aim:** The aim of this article is to review the literature on the use of antimicrobial additives in glass-ionomer dental cements.

**Method:** An electronic search between 1987 and the end of 2017 was performed using PubMed, Web of Science and Google search engines with the terms glass-ionomer, glass polyalkenoate, antibacterial and antimicrobial as the key words. The search was refined by excluding the majority of references concerned with cement antimicrobial properties only. Extra papers already known to the authors were added to those considered.

**Results:** A total of 92 relevant articles have been cited in the review of which 55 are specifically concerned with the enhancement of antibacterial properties of glass-ionomers, both conventional and resin-modified, with additives. In addition, information is included on the uses of glass-ionomers and the biological properties of the antibacterial additives employed. There are several reports that show that additives are typically released by diffusion, and that a high proportion is usually left behind, trapped in the cement. Additives generally increase setting times of cements, and reduce mechanical properties. However, smaller amounts of additive have only slight effects and the longer-term durability of cements appears unaffected.

**Conclusion:** Modified glass-ionomer cements seem to be acceptable for clinical use, especially in the Atraumatic Restorative Treatment (ART) technique.

## Introduction

Glass-ionomer cements are acid-base materials that are widely used in clinical dentistry [1]. Applications include full restorations, particularly in children, liners and bases, fissure sealants, luting agents and also, to a lesser extent, adhesives for orthodontic brackets bands [2] and as endodontic sealers [3].

Typically, glass-ionomers consist of a fine powder of basic glass and a solution of polymeric acid, such as poly(acrylic acid) in water [1]. These formulations are considered to be conventional glass-ionomers and they set by an acid-base reaction that results in the formation of a polysalt. The glass is a complex material that consists of calcium or strontium alumino-silicates, together with added phosphate and fluoride components [4]. Its basic character is controlled by the ratio of alumina to silica in the glass formulation and is designed so that the finished glass powder can react with the polymer solution to form a hardened material in about 2–3 min.

In the early 1990s, the resin-modified glass-ionomer was introduced to the dental profession [5]. The essential feature of this material is that, as well as the components of the conventional glass-ionomer, it contains a monomer and an initiator system. On irradiation by visible light from a dental cure lamp, the initiator triggers polymerization of the monomer. This cement therefore sets by dual mechanisms, namely polymerization and neutralization. This results in a complex set material, and physical properties depend to an extent on the time between mixing, which begins the neutralization, and polymerization [6]. The monomer used in these cements is 2-hydroxyethyl methacrylate, HEMA [5]. HEMA is water-miscible, but poly(HEMA) is insoluble in water. Despite this, no phase-separation occurs, and the cement sets to give a uniform material whose physical properties closely resemble those of the conventional glass-ionomer.

Glass-ionomer cements of both types are used to repair teeth damaged by caries. Caries is known to result from metabolic activity of certain microorganisms on the surface of the teeth, the most significant of which is *Streptococcus mutans* [7]. The fact that such bacteria may remain on the tooth surface to which a restorative material is applied [8–10] has led to the suggestion that materials with antimicrobial properties may be beneficial. In the case of glass-ionomers, the materials show a degree of antibacterial character, but this may need to be augmented by adding bactericides to increase the therapeutic benefit.

**Figure 1.**
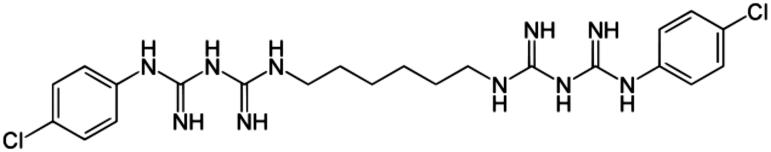
Chorhexidine.

**Figure 2.**
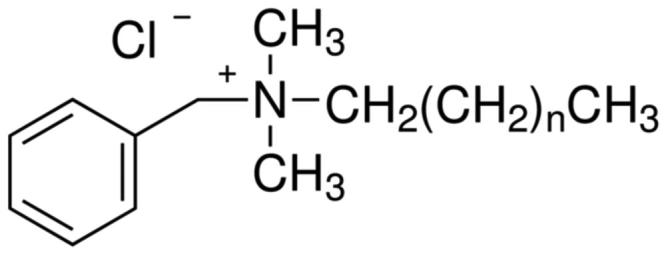
Cetyl pyridinium chloride.

**Figure 3.**
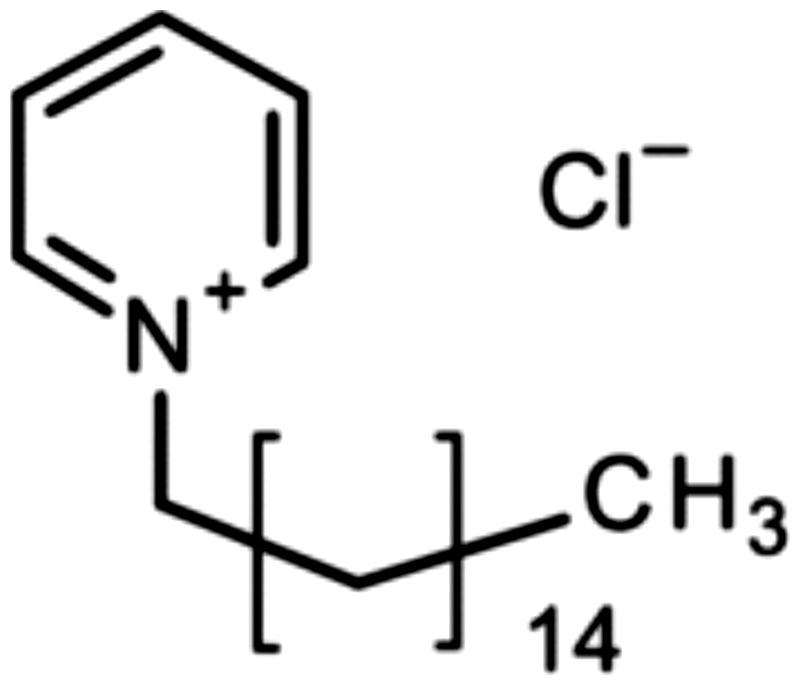
Benzalkonium chloride.

**Figure 4.**
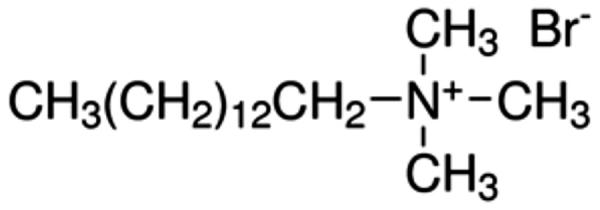
Cetrimide.

Several antibacterial substances have been studied for use as additives within glass-ionomer cements. These substances must to be carefully selected, because they must not be toxic towards the cells of the pulp or the gingiva but must still be able to protect against the growth of cariogenic bacteria [11]. A search of the literature has shown that a small group of organic substances have been studied in detail by several workers, namely chlorhexidine (solubilised as either as the diacetate or the digluconate), cetylpyridinium chloride, benzalkonium chloride and cetrimide ( Figures 1–4). Other substances, including inorganic compounds [12–14], have been used, but studies on them are more limited.

The present review concerns various aspects of the use of these antimicrobial additives in glass-ionomer cements. It has been compiled by studying literature published between 1987 and the end of 2017. Relevant papers have been identified electronically using PubMed, Web of Science and Google search engines with the terms *glass-ionomer*, *glass polyalkenoate*, *antibacterial* and *antimicrobial* as the key words. This search identified a number of studies that were concerned with the inherent antibacterial or antimicrobial properties of the cements themselves, and not with the inclusion of additives. These references have not been included in the current review; by contrast, all references reporting studies on the effect of antibacterial additives in glass-ionomers have been included. In addition to those papers identified electronically, a small number of extra papers already known to the authors were added to those considered. The overwhelming majority of reports have been found to be *in vitro* studies and, despite claims made for potential effectiveness of the approach, few *in vivo* studies and no full clinical trials have been reported on glass-ionomers containing antimicrobial additives.

## Controlled release from conventional glass-ionomers

The first study of the release of organic antibacterial substances appeared in 1991, and used chlorhexidine diacetate [11]. Two concentrations were used (13.3% and 6.65%) with AquaCem (Dentsply), a commercial water-activated luting cement. Results showed that antimicrobial properties improved [11]. Glass-ionomers have some slight antimicrobial properties, even without additive, as a result of their fluoride release [15,16]. This was shown by Seppa et al. [17], with glass-ionomers against *Streptococcus mutans*. It has also been shown generally with glass-ionomers against plaque [15]. This is attributed to fluoride release, a feature which may protect teeth from secondary caries [18].

Recently, the antimicrobial properties of three conventional glass-ionomers against three species of cariogenic bacteria have been reported [19]. The bacteria were *S. mutans*, *S. oralis* and *S. salivarius*, and experiments were performed using a diffusion method on a solid medium. Antibacterial activity was determined after 48 h by measuring the size of the halos of growth inhibition around the specimens of set cement. Cements showed significant inhibition, which confirmed their inherent antimicrobial character, due to their fluoride release and low pH immediately after placement [20–22].

The focus of the early work was on the antimicrobial effects, and it was not until several years later that Palmer et al. [23] published a study that considered the mechanism of release, the setting chemistry with additives and the resulting physical properties when set. The study used an experimental cement of known composition [24] containing between 0.4 and 135 by mass chlorhexidine diacetate. After curing, release of chlorhexidine diacetate was determined at regular intervals using reverse-phase high-performance liquid chromatography (HPLC).

There was a general pattern that both working and setting times increased with increasing amounts of chlorhexidine diacetate. For example, additive-free cement had a working time of 2.8 min, which rose to 3.6 min with 11.44% chlorhexidine diacetate. As well as taking longer to set, cements with additive were weaker (Table 1). There was a linear relationship between amount of chlorhexidine diacetate in the cement and the compressive strength, and with no additive, the compressive strength was 226.8 MPa whereas with 11.28 chlorhexidine diacetate, strength fell 136.3 MPa.

**Table 1.** Effect of antimicrobial additives on the setting and strength properties of glass-ionomer cements.

Cement	Additive	Amount/%	Working time/min	Compressive strength, 24 h/MPa	Ref.
Experimental	Chlorhexidine diacetate	11.30/11.44	2.8	269.0	[23]
			3.6	125.5	
Chemflex	Chlorhexidine diacetate	0	2.4	221.1	[22]
		0.5	2.4	213.6	
		1.25	2.5	175.1	
		2.50	2.5	177.9	
Chemflex	Chlorhexidine digluconate	0.5	2.6	219.8	[22]
		1.25	2.6	207.6	
		2.50	3.2	211.6	
Chemflex	Benzalkonium chloride	1.0	5.0	129.6	[24]
		2.0	5.8	122.0	
		3.0	5.8	96.6	
Chemflex	Cetylpyridinium chloride	1.0	4.5	101.7	[24]
		2.0	4.8	73.4	
		3.0	3.8	66.4	

All cements formulated with chlorhexidine diacetate released additive, with the early part of release being a function of √t, a feature characteristic of a diffusion mechanism [25]. Plots remained linear for between 7 days and 20 days, depending on the amount of additive in the cement. In all cases, considerable chlorhexidine diacetate was retained after 240 days (between 90 and 97% of the total). The lowest retention (90%) occurred with the highest loading of chlorhexidine diacetate, and the other levels gave greater retention values, i.e. between 95 and 97%. The authors suggested that chlorhexidine diacetate becomes bound within the cement, either chemically or physically, and that release occurs until only the unbound fraction has been released.

Some of the changes in release are due to the influence of the chlorhexidine diacetate on the setting reaction of the cement [23]. This interference is shown by the lengthening of the working and setting times (Table 1). Reduction in setting rate typically leads to weaker cements, evidence that the matrix is mechanically deficient and different from the matrix that forms with no additive. Recently Dimkov et al. [24] came to similar conclusions, using benzalkonium chloride and cetylpyridinium chloride. Setting times increased and set cements were weaker when additives were present [24], and these observations have been confirmed in several other studies.

Botelho published two papers in which the effect of antimicrobial compounds in glass-ionomer cements was studied in depth [26,27]. The first paper [26] reported on the inhibitory effects of four cationic compounds, namely chlorhexidine hydrochloride, cetylpyridinium chloride (Figure 2), benzalkonium chloride (Figure 3) and cetrimide (Figure 4), added to the conventional glass-ionomer cement Fuji IX at 1, 2 and 4% by mass. Results were compared with control samples of cement that contained no additive. Experiments used the agar diffusion test, with specimens of cement placed onto agar plates inoculated with an appropriate species of bacteria [26]. Two species each of *Streptococcus*, *Lactobacillus* and *Actinomyces* were used, making a total of six different types of bacteria. The area of inhibition was determined after 24 h, then at weekly intervals. At week 11, the surfaces of the cement specimens were abraded, then replaced onto the inoculated agar plates and left for a further week.

Under these experimental conditions, the additive-free specimens of Fuji IX showed no antibacterial effect at all [26], which is surprising, especially in the earliest time periods when fluoride release would be expected to be at its highest. By contrast, all of the specimens containing antimicrobial compounds were antibacterial, with extent varying with the amount of additive. In all cases, this effect diminished over time, until week 11, when abrading the specimen surfaces led to marked increases in release [26]. Cetrimide was the most effective antimicrobial compound against four of the six species of bacteria tested [26].

The second part of the study concerned the effect of the antimicrobial additives on the compressive strength of the cement [27]. The results were similar to those obtained by Palmer et al. [23] in that cements containing additives had significantly reduced compressive strengths in all cases except for 1% benzalkonium chloride, where strength was not significantly different from that of the control cement. In all cases, increasing additive level caused greater reductions in the compressive strength. The study concluded that the adverse effect on physical properties could potentially affect the clinical performance of the glass-ionomer cement [26]. Unlike the study of Palmer et al. [23], this study did not include any measurement of the rate of setting, so there was no information on how these additives affect the acid-base reaction.

Antibacterial effects of adding benzalkonium chloride and cetylpyridium chloride in conventional glass-ionomers were confirmed by Dimkov et al. [24,28]. Additives were incorporated at levels of 1, 2 and 3% by mass into the commercial glass-ionomers Chemflex (Dentsply, Germany) and Fuji IX (GC, Japan). Cylinders of set cement (4 mm diameter ×6 mm height) were used in an agar diffusion test against *Streptococcus mutans*, *Lactobacillus casei* and *Actomyces viscosus* [29]. Inhibition zones were measured after 2, 7 and 21 days. In all cases, the zones of inhibition were larger than those around the cement with no additive [28]. Also, zones became slightly smaller over longer time periods. Benzalkonium chloride was more potent against all three species of bacteria than cetylpyridinium chloride, but all results confirmed that glass-ionomer cements are able to act as the medium for the slow release of antibacterial compounds [28].

The microbiological effects of adding chlorhexidine digluconate to conventional glass-ionomer cements have also been reported [30]. This substance is effective against both Gram-positive and Gram-negative bacteria, even when employed at high dilutions [31, 32]. The study [30] used two conventional glass-ionomer cements, namely Fuji II and Fuji IX (both GC, Japan), with chlorhexidine digluconate added to the glass-ionomer liquid in the mass ratio 0.5:9.5, to give concentration of 5% (mass/mass).

Testing used cylindrical specimens of cement (5 mm diameter ×11 mm height) in an agar diffusion test against *S. mutans*. Inhibition zones were measured at 1, 7 and 14 days. At all times, specimens showed substantial inhibition zones, with size decreasing slightly with time (Table 2). Fuji II gave greater sized zones, a finding that was linked to the fact that it contained less glass than Fuji IX, and consequently set more slowly. This led to the formation of cements that released chlorhexidine digluconate more readily than the denser, faster setting Fuji IX [30].

**Table 2.** Zones of inhibition (mm) around glass-ionomers loaded with and without chlorhexidine digluconate (Standard deviations in parentheses) [30].

Cement formulation	1 day	7 days	14 days
Fuji II only	15.00 (0.67)	11.00 (0.00)	11.00 (0.00)
Fuji II + CG	25.50 (1.27)	23.520 (1.23)	20.20 (1.32)
Fuji IX only	13.60 (0.52)	11.00 (0.00)	11.00 (0.00)
Fuji IX + CG	24.70 (0.95)	22.80 (0.63)	19.70 (1.25)

The effects of antimicrobial compounds in conventional glass-ionomer cements was reported by Turkun et al. in 2008 [31], employing both chlorhexidine diacetate and chlorhexidine digluconate, and incorporating them into the commercial cement ChemFil Superior (Dentsply De Trey, Germany). Additions were made respectively to the powder (chlorhexidine diacetate) and the liquid (chlorhexidine digluconate) at overall concentrations of 0.5, 1.25 and 2.5% within the cement. Various properties of the resulting cement were tested (setting time, working time, compressive strength, acid erosion, diametral tensile strength and biaxial flexure strength) and long-term antibacterial behaviour against *S. mutans*, *L. acidophilus* and *C. albicans* were evaluated using the agar diffusion test.

For this cement, working and setting times, erosion by acid, diametral tensile strength and biaxial flexure strength were not affected by the presence of the additives at any concentration (Table 1). Results were not significantly different from those obtained for the additive-free control sets of ChemFil Superior. However, both compressive strength and hardness were affected in some cases: the 1.25% and 2.5% chlorhexidine diacetate groups had significantly lower compressive strengths, and the 0.5 and 2.5% chlorhexidine digluconate groups had lower hardness [27]. In all cases, antibacterial properties were improved by the additives, with the greatest effects occurring at higher concentrations (2.5% for both additives).

The fact that both additives had little effect on the physical properties of the cement while improving its antibacterial character is important. It suggests that, as low levels, antimicrobial additives are almost completely beneficial. The authors concluded that further studies of ChemFil Superior with antibacterial additives were needed to determine whether these formulations do, indeed, have any advantages when used in patients [31].

Another study, this time using chlorhexidine digluconate in Ketac Molar Easymix (3M ESPE, Germany), came to similar conclusions [33]. Data from this study are also shown in Table 3, and both the setting time and the surface hardness declined with greater amounts of additive. Inhibition zones were determined for both *S. mutans* and *L. casei*, and no significant differences were found with increased loadings though there was no inhibition at all with the additive-free cement. Also, the inhibition zones were greater for *L. casei* than for *S. mutans* [33].

**Table 3.** Effect of including chlorhexidine digluconate (CHX) in Ketac Molar Easymix (3M ESPE, Germany) [34].

Cement formulation	Setting time/min	Surface hardness/VHN	Tensile bond strength/MPa	Zone of inhibition, *L. casei*	Zone of inhibition, *S. mutans*
KME only	5.15	33.40	10.46	0	0
KME +0.5% CHX	6.10	32.91	8.05	15.07	13.85
KME +1.0% CHX	6.65	27.89	8.33	15.11	13.74
KME +2.0% CHX	10.15	14.41	5.65	15.45	14.21

Similar results were found for chlorhexidine digluconate in the cement Ketac Molar Easymix [34]. This material is suitable for use in the Atraumatic Restorative Treatment (ART) technique, and the potential of the modified material for use in ART was the focus of the study. It employed the additive at 1.25% and 2.5% by mass without any other alterations to the powder:liquid ratio. Antimicrobial properties were evaluated in the agar diffusion test against *S. mutans*, *L. acidophilus* and *C. albicans*. Compressive strength and Knoop hardness were also tested. At 1.25%, chlorhexidine digluconate did not affect the mechanical properties or fluoride release, but improved the antibacterial effects. By contrast, at 2.5% the mechanical properties and the fluoride release were both affected adversely. As a result, the 1.25% level was recommended for clinical use in ART [34].

The paper also reported a clinical evaluation of Ketac Molar Easymix with 1.25% chlorhexidine digluconate [34]. This involved restorations in 136 children aged 3–6 years, and showed that after 1 year, durability was not affected by the presence of the additive.

A study carried out using both a conventional and a resin-modified glass-ionomer cement showed that chlorhexidine diacetate caused significant inhibition of bacterial vitality and biofilm formation *in vivo* [35]. Samples of cement with and without additive (control) were bonded to the buccal surfaces of molars in the first and second quadrant of volunteers. They were left for time periods of 4 and 24 h respectively then the bacterial vitality of the plaque was analysed by confocal laser scanning microscopy. Bacterial morphology and biofilm accumulation were determined by scanning electron microscopy. Results showed that the bacterial vitality with the additive was lower than on the control materials and also no effect was observed on surface hardness, despite the relatively high loading of 2% by mass that was used.

The effect of additives on fluoride release was studied using higher loadings compared with most other studies, i.e. 10% by mass chlorhexidine digluconate [36]. Results showed that fluoride release was reduced in the presence of additive. This work used the cement AquaCem (Dentsply, Germany) and results showed that the antimicrobial properties were improved, despite the reduction in fluoride release [36].

Studies typically report the use of a single antibacterial compound in glass-ionomer cements. However, two publications [37,38] have dealt with the inclusion of a pair of additives, namely chlorhexidine diacetate and cetrimide, both at 2.5% by mass. In the first report, the combination was used to improve antimicrobial properties of two restorative grade glass-ionomers (Fuji IX and Ketac Molar), and the combined additives were incorporated into the glass powder prior to mixing the cement. Set cements were tested in an agar diffusion test against *S. mutans* and *L. casei* at times between 1 and 90 days. Vickers Hardness number was determined after 1 day. Results showed that this combination of additives improved the antimicrobial properties of the cements with only minor effects on the hardness. The authors concluded that resulting cements were suitable for use with ART procedures [37].

The same additives were tested in various acid-base luting cements (conventional glass-ionomer, zinc polycarboxylate and zinc phosphate) [38]. Again, antibacterial properties were evaluated in an agar diffusion test against *S. mutans* and *L. casei*, this time for 180 days. Flexural strength and solubility were also determined, and the strength was found to be reduced while the solubility increased. Despite these adverse effects, the authors considered the use of this combination to be useful, since it has the potential to reduce or eliminate bacteria in inaccessible places, such as beneath cemented crowns in repaired teeth [38].

## The effect of organic additives on conventional glass-ionomer cements

The effect of organic antibacterial additives on the properties of the cement have been widely studied [22,23,27,39]. Data from various studies are shown in Table 1. These show that additives have two effects, namely slowing down the setting reaction and reducing the mechanical properties of the cement. Similar findings have been reported for both neutral organic additives and for ionic compounds. For example, the organic compounds methanol and 2-hydroxyethyl methacrylate (HEMA) both reduced the speed of the setting reaction and also the compressive strength at 24 h [40] (see Table 4).

**Table 4.** Effects of organic additives on a glass-ionomer cement [40].

Cement	Additive	Amount	Working time/min	Compressive strength, 24 h/MPa
Aquacem	Water-activated only	0	3.6	230
	Methanol	1:1 (v:v)	12.8	170
	2-hydroxyethyl methacrylate	1:1 (v:v)	6.6	147

Methanol causes poly(acrylic acid) molecules to adopt more coiled conformations than they do in pure water [41] and 2-hydroxyethyl methacrylate probably has a similar effect [40]. The more tightly coiled the polymeric acid molecules are, the harder they are to ionize. This, in turn, reduces the acidity and consequently slows down the setting process.

The effect of ionic compounds on poly(acrylic acid) molecules is more complicated [42,43]. Ionic compounds, such as sodium chloride or potassium bromide, are typically added to very dilute solutions of polyelectrolytes such as poly(acrylic acid) to cause coil expansion when determining molar mass by viscosity measurements. Whether they have the same effect on coil dimensions at the type of high concentrations of poly(acrylic acid) used in glass-ionomer cements is debatable. However, these additives do have effects on the properties of these cements, as the data in Table 5 demonstrate.

**Table 5.** Effect of ionic compounds in 1 mol dm^−3^ solutions on the setting and strength properties of water-activated glass-ionomer cements [4,422].

Cement	Additive	Working time/min	Compressive strength, 24 h/MPa
Aquakent	None	3.6	95.2
	NaCl	3.3	87.2
	NaF	4.1	89.2
	Na_2_SO_4_	4.7	56.8
Aquacem	None	4.2	94.3
	NaCl	4.2	59.8
	KCl	4.4	65.8
	KBr	4.2	67.0

The results shown here were obtained with brands of water-activated glass-ionomer cement, AquaKent (Kent Dental, UK) and AquaCem (Dentsply, Germany) that are usually prepared by reacting the powder with pure water, but in this case were activated with aqueous solutions of ionic compounds at a concentration of 1 mol dm^−3^. This suggests that at the concentrations of poly(acrylic acid) in the cement, the salts induce conformational changes. The presence of salts has also been shown to increase the pH of aqueous solutions of poly(acrylic acid), which confirms that the dissolved salts provide electrostatic shielding and thereby stabilize the charge-separated form of the polymeric acid. This increases the concentration of hydrogen ions, hence decreasing pH. For example, 1 mol dm^−3^ aluminium nitrate solution reduced the pH of poly(acrylic acid) solution from 1.5 to 0.2 [44].

It is not clear why this greater acidity should lead to a slower setting reaction, but the experimental results show that it does. Slower setting leads to weaker cements though the reason for this is not clear, despite it being well established.

In another study, Tüzüner and Ulusu studied the surface hardness of Fuji IX specimens with and without antibacterial additives [45]. They used cetrimide (CT), cetylpyridinium chloride (CPC), chlorhexidine (CHX) and benzalkonium chloride (BC) at levels corresponding to 1% and 2% by mass. The first three were added to the glass powder, while benzalkonium, because of its hygroscopic character, was added to the liquid.

The study evaluated hardness using the Vicker’s Hardness Number, VHN. This property was studied because of its importance in controlling resistance to wear [46], and results give a quantitative indication of the potential durability of these cements. In their experiments, Tüzüner and Ulusu measured VHN after storing cement specimens in distilled water for time periods of 1, 7, 15, 30, 60 and 90 days [45].

The results showed statistically significant differences in VHN between the controls and the experimental cements for all time periods [45]. Trends over time were different, with additive-containing specimens showing decreased VHN with time, whereas in the control groups, VHN increased with time. This suggests that the additives interfere with the maturation of the cement, as well as the setting reaction. Additives did not all perform the same way. Benzalkonium chloride and chlorhexidine had the least negative effect, whereas cetrimide and cetylpyridinium chloride had the most negative effects on VHN.

Tüzüner and Ulusu concluded that, despite the reductions in VHN, groups containing additives had acceptable properties throughout the experimental time period. They therefore suggested that, despite their study being limited to *in vitro* results only, these additives could be used clinically in Fuji IX, for example in the ART technique [45]. However, in view of the greater effects of cetrimide and cetylpyridinium chloride, these additives were recommended for use at lower concentrations, i.e. 1% rather than 2%.

The marginal seal in restorations with additives was considered in an *in vivo* study involving the cement Fuji IX with 1% chlorhexidine diacetate placed in healthy molars in children [46]. Teeth were restored with either the cement alone or cement with additive, then extracted at 4 weeks and leakage detected by storing the teeth in basic fuchsin solution for 24 h. Teeth were sectioned and examined by light microscopy. No differences in microleakage were found between the two groups, showing that 1% chlorhexidine digluconate would not compromise the sealing abilities *in vivo* [47].

## Studies with natural products

The majority of papers on controlled release from glass-ionomer cements concentrate on a limited range of antimicrobial compounds. However, some other substances have been studied. For example, the combination of casein phosphopeptide/amorphous calcium phosphate with lysozyme, lactoferrin and lactoperoxidase (LLL) added to a glass- ionomer cement was then used to restore extracted third molars in an *in vitro* study [48]. These teeth were then exposed to a standard strain of *S. mutans* and results showed that there was a significant reduction in numbers of *S. mutans* with LLL only at 1 month, though numbers increased by 6 months. The 1 month reduction in bacterial growth was considered clinically desirable, as it would inhibit the progress of caries in newly restored teeth [48]. Unfortunately, no results were reported on how the LLL combination altered setting or strength properties.

**Figure 5.**
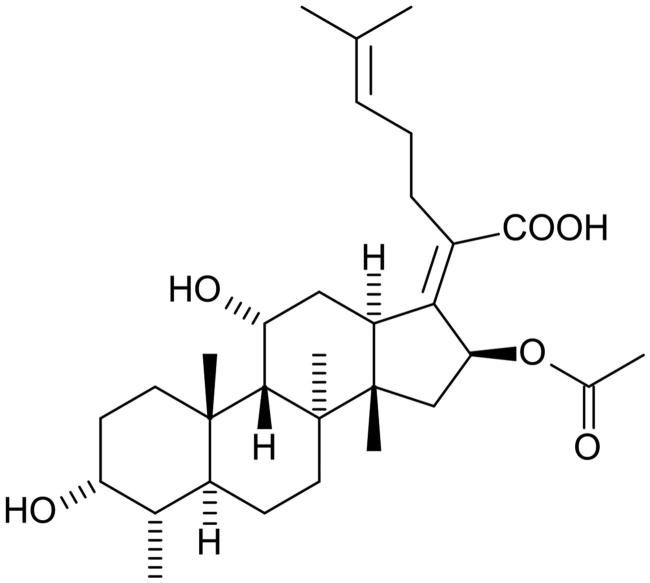
Fusidic acid.

In another *in vitro* study, the effect of including fusidic acid (Figure 5) in a conventional glass-ionomer cement was reported [49]. Fusidic acid was chosen because of its effectiveness against staphylococcus infections [50], suggesting it might have a role in protecting against bacterial infection when glass-ionomer cements are used in bone repair surgery [49]. Fusidic acid is obtained from the fungus *Fusidium coccineum* and is widely used to treat skin infections. It interferes with protein synthesis in the target microorganisms [51], though there are problems because these organisms are developing resistance [52].

Fusidic acid release from a glass-ionomer cement was measured using reverse-phase HPLC and followed a diffusion mechanism. It was added at levels of 1% and 5% by mass, with little effect on either diffusion coefficient or proportion released. The latter value ranged from 20 to 23% after 2 weeks, compared with only about 4% for benzalkonium chloride [26]. This showed that, despite its bulk, the fusidic acid molecule is released more easily than the quaternary ammonium salt. Unfortunately, this study, too, failed to carry out experiments on either setting rates or compressive strength of set cements. As a result, it is not clear how effective the approach of adding fusidic acid might be in practical glass-ionomer cements.

A number of other naturally occurring substances and mixtures are known to be anti-bacterial and some of these have been studied as possible additives to glass-ionomer cements. For example, the natural polyphenol in green tea, epigallocatechin-3-gallate (ECGC) has been used in this way [53]. EGCG has known anticaries properties [54] due to its ability to suppress amylase activity in both saliva and bacteria, and thereby reduce the rate of carbohydrate metabolism. However, effects of incorporating EGCG into glass-ionomer cement were limited.

In the study, EGCG was added at a level of 0.1% by mass to the cement [53]. At this level of addition, both the flexural strength and the anti-bacterial properties of the modified glass-ionomer were improved at 4 h, but any such effects had disappeared by 24 h. There was evidence of release of EGCG to bring about the improvements in anti-bacterial character, but this was not sustained at a sufficient level to have an effect by 24 h [53]. So far, there have been no further studies of the use of this substance in glass-ionomers, though there might be benefits in adding EGCG at higher levels and determining the effects of this on the mechanical properties as well as the antibacterial characteristics.

Another natural substance that has been used in this way is propolis. This is a resinous material produced by bees and has widespread medical use on account of its antibacterial properties [55]. Studies have shown that the addition of propolis to glass-ionomer cements enhances their antibacterial properties [29,55,56]. This substance is particularly active against cariogenic bacteria of the *Streptococcu*s genus, in particular *S. mutans* and *S. sobrinus* [57]. Though propolis has some adverse effects on the mechanical properties of glass-ionomers, for example reducing the compressive strength slightly, it was shown to improve the micro-hardness and had no adverse effect on micro-leakage [58]. Again, this substance appears to merit further investigation in this application.

Lastly, *Salvadora persica* extract (SPE) has also been used as an antibacterial additive in glass-ionomers [59,60]. The addition of 4% SPE to glass-ionomer led to improvements in their antibacterial properties, notably against *S. mutans* and also against *S. sangus* and *Candida albicans*. Physical properties of the modified cements were affected only slightly by the presence of SPE, and remained comparable with other commercial glass-ionomer cements.

## Inorganic anti-microbial additives

Some commercial glass-ionomers are made from strontium-containing glasses where the element strontium effectively replaces calcium in the structure [1]. As such, it is insoluble under neutral conditions but is released from cements under acidic conditions. Strontium has been studied for its antibacterial properties [61,62] and results show that its presence enhances the anti-bacterial activity of glass-ionomer cements to a substantial extent [63]. Details of its mechanism are not known, and the sensitivity of its release to the surrounding pH may limit its usefulness in clinical application.

Glass-ionomers themselves have been developed to have increased antibacterial character to enable their use in non-dental applications, such as orthopaedics or cranioplasty [64–66]. These glasses contain no aluminium, so that there can be no release of Al^3+^ ions, species which have been implicated in degenerative brain disease [66]. Instead, they are zinc-based. They are capable of forming cements with aqueous solutions of polyacrylic acid [65] and the resulting cements have been shown to release antimicrobial Zn^2+^ ions in water and simulated body fluid [65,67]. The majority of this release has been found to occur within the first 24 h [65], which may be sufficient to provide an antibacterial environment for the chosen applications [68]. Inclusion of silver in these glasses has also been found to improve the antibacterial efficacy of the resulting cements, at least for the first 24 h or so, due to leaching of both Zn^2+^ and Ag^+^ ions [68,69]. To date, glass-ionomer cements based on these zinc glasses have been studied as experimental materials only, and there have been no reports of any clinical studies involving their use. Anyway, they are designed for very different applications from dentistry, and for this reason they are not considered further in this review.

Zinc has also been added to dental-grade glass-ionomer cements as the sulfate salt [70]. This compound is readily soluble under neutral conditions, so that zinc ions are released steadily into the surroundings, thereby enhancing the antimicrobial properties of the glass-ionomer cement.

Zinc oxide nanoparticles have been employed in glass-ionomer cements [71,72]. Zinc oxide is much less soluble that zinc sulphate, particularly under neutral conditions, so this approach is less effective in providing Zn^2+^ ions than using zinc sulfate. Nonetheless, ZnO nanoparticles have been found to make at least some improvements in the antibacterial character of cements containing them [71,72]. Issues of safety of nanoparticles were not discussed in this article, though they would need to be considered before the wider public would accept such formulations for dental repair.

Silver nanoparticles have also been added to glass-ionomer cements in order to enhance their antibacterial properties [73]. Cements containing these silver nanoparticles were tested in an agar diffusion test, and showed substantially increased zones of inhibition. This suggests that there had been a significant transport and release of silver ions from these cements. However, this improvement in antibacterial character was not sustainable, probably because of the rapid depletion in silver and also of fluoride in the cements. Because of this, zones of inhibition were almost absent in specimens aged for 2 days or more. This suggests that the addition of silver nanoparticles is unlikely to be useful clinically. The paper also did not address any safety concerns with the nanoparticles used, though in view of the outcomes of the experiments, this is not a serious failing.

Nanoparticle titanium dioxide has been incorporated into glass-ionomer cements and found to improve their mechanical properties [74,75]. At levels of 3, 5 and 7% (w/w) these nanoparticles have also been found to prevent growth of bacteria [74]. Since the level of fluoride release in cements containing TiO_2_ was not affected by the presence of the additive, it follows that the TiO_2_ itself must have imparted antibacterial properties on to the cements. However, the mechanism is not clear, as TiO_2_ is of generally low toxicity and the presence of titanium dioxide on the surface of titanium alloy implants is generally considered a positive aspect, contributing to their biocompatibility in bone contact [76].

## Controlled release from resin-modified glass-ionomers

There have been fewer studies on adding antimicrobial compounds to resin-modified glass-ionomers than to conventional glass-ionomers. An early study used chlorhexidine diacetate (5% concentration) in the resin-modified glass-ionomer Photac-Fil (3M, USA) [77]. Samples were tested for hardness, diametral tensile strength and erosion levels at 24 h and 6 weeks. Chlorhexidine diacetate elution was determined at weekly intervals, and antibacterial properties were measured at 6 weeks only. No differences were found in diametral tensile strengths for specimens containing the additive at either 24 h or 6 weeks. Hardness also did not differ at 24 h, but had become significantly lower for the samples containing chlorhexidine diacetate after 6 weeks. Results from erosion studies showed that the chlorhexidine group lost less material than the additive-free control at 24 h, but significantly more at 6 weeks. Elution levels were highest at 1 week, and substantial antimicrobial effects were recorded against *S. mutans*. Similar antibacterial properties were found at weeks 2 and 3, but not afterwards.

The authors concluded that chlorhexidine diacetate improved the antimicrobial behaviour of this resin-modified glass-ionomer without seriously affecting its physical properties. However, no conclusions could be drawn about long-term clinical performance [78].

Chlorhexidine digluconate has also been used as an antimicrobial additive in resin-modified glass-ionomers [54]. This substance was added to the commercial resin-modified glass-ionomer Fuji Lining LC (GC, Japan) at levels of 0.2, 0.5, 1.25 and 2.5% by mass. As before, results were compared with those of control specimens of the cement containing no additive. Antimicrobial properties were determined using an agar diffusion test against *S. mutans*, *L. acidophilus*, *L. casei* and *A. viscosus* as test organisms [54]. Tests were also carried out using immortalized odontoblast-like cells (MDPC-23) and cell metabolism was analysed using MTT assay. Mechanical properties (compressive and diametral tensile strength) of cement specimens were also measured. These tests were carried out on specimens that had been stored in distilled water at 37 °C for 24 h.

Results showed, not surprisingly, that the most effective antimicrobial effects were obtained with the highest levels of addition of chlorhexidine digluconate (1.25% and 2.5%) [77]. This applied to all test organisms, though there were differences between species. *S. mutans* was found to be the most susceptible to inhibition by the additive at all concentrations. These results were supported by those from the tests on cell metabolism, which confirmed that, at 2.5%, chlorhexidine digluconate in resin-modified glass-ionomer caused a significant reduction in the metabolic activity of the MDPC-23 cells. Some change in morphology was also observed in cells exposed to 2.5% chlorhexidine digluconate. Findings from mechanical tests were similar to those obtained for conventional glass-ionomers, specifically that diametral tensile strength was not affected but that, with 2.5% chlorhexidine digluconate, compressive strength was significantly reduced.

The authors of this paper went on to carry out a limited *in vivo* study using the resin-modified glass-ionomer with 1.25% chlorhexidine digluconate in clinical procedures that involved only partial removal of caries [78]. This involved a total of 13 teeth (7 control, 6 with additive-containing RMGIC) from ten patients who were children aged 4–9 years. Treatment involved indirect pulp treatment with the materials (control or additive-containing) followed by re-examination within 3 months. Re-examination involved re-opening the tooth and removing the restorative and liner materials carefully and completely. These were then examined for bacterial content. Results showed that there were substantially fewer microorganisms on samples collected from teeth that had been restored with chlorhexidine-containing cement compared to those of the control group. This finding confirms that the presence of such an antibacterial additive may function to eliminate residual microorganisms below the material after indirect pulp treatment. The authors therefore concluded that the addition of chlorhexidine digluconate to the cement used for repair is a useful therapeutic strategy in caries management [78].

## The antibacterial additives and their biological effects

The majority of antibacterial substances used in glass-ionomer cements are ampiphilic compounds with a reasonable degree of surface activity. This includes both the chlorhexidine species used (diacetate and digluconate) as well as benzalkonium chloride, cetylpyridinium chloride and cetrimide [54,79]. These substances are broad-spectrum antimicrobials that are particularly effective against Gram-positive bacteria. They are used as antiseptics and antimicrobials, including in oral hygiene products such as mouthwashes and lozenges [80,81].

They function by disrupting the cell membranes in target bacteria [54,82] leading to cell lysis and death [83]. For a long time it was thought that microorganisms could not develop resistance to this mechanism but this is now known not to be the case. In recent years microbial resistance to compounds such as benzalkonium chloride has been demonstrated experimentally [83,84].

Resistance develops through the development of structures inside the cells that act as efflux pumps and expel the antimicrobial compound [54,85]. Other mechanisms have been identified, such as modifications to the membrane, changes in stress response and improvements in repair systems [86]. This has led to concerns that the widespread use of compounds of this type as antimicrobial additives in cosmetic products is likely to increase the selection pressure and encourage more resistant strains of microorganisms to develop [86].

As previously mentioned, the principal microorganism implicated in dental caries is *S mutans* [87,88]. This is a Gram-positive bacterium that occurs in the human mouth and has been shown to be able to develop resistance to a number of antibiotics, such as penicillin and tetracycline [88]. It an also develop resistance to the antimicrobial effects of fluoride [89]. However, to date there have been no reports of this species developing resistance to any of the quaternary ammonium compounds studied in glass-ionomers (i.e. chlorhexidine diacetate and digluconate, cetrimide, benzalkonium chloride and cetylpyridinium chloride). One study has specifically shown that, even after ten passages, *S. mutans* showed no increase in resistance to cetylpyridinium chloride or chlorhexidine [90]. By contrast, the bacterium *Enterococcus faecalis*, on exposure to these substances, developed more hydrophobic cell surfaces and became more resistant to chlorhexidine [90]. At the moment, therefore, it seems that most of the antimicrobial compounds employed in glass-ionomers are remaining effective against the main target microorganism, *S. mutans*.

This observation is also true for triclosan. It operates differently from the quaternary ammonium compounds in that it inhibits fatty acid synthesis within bacterial cells [91]. Like the quaternary ammonium compounds, it is employed in various consumer products, such as soaps and detergents, and also toothpastes [92]. Some bacterial species have been found to develop resistance to triclosan, notably *Escherichia coli* and *Salmonella enterica* [92], but so far no such resistance has been reported for *S. mutans*.

## Conclusions

This review has shown that there are potential clinical advantages in adding antimicrobial compounds to glass-ionomer dental cements, either conventional or resin-modified. A limited number of substances have been studied in depth, namely chlorhexidine diacetate, chlorhexidine digluconate, benzalkonium chloride, cetylpyridinium chloride and cetrimide, though others, including natural products and inorganic substances, have also been considered and reported in the literature. Typically such substances reduce the mechanical properties of the cements, though higher loadings have more effect; these additives may also reduce fluoride release. Release is typically a diffusion processes, and at amounts that show effects against bacterial population. Longer-term studies are currently lacking, and further work is necessary to confirm the effectiveness of this approach. The particular organic compounds that have been used as additives do not lead to resistance in the target microorganism *S. mutans*, so this approach has potential clinical advantages for use in patients.
